# Biomimetic Nanomaterials Triggered Ferroptosis for Cancer Theranostics

**DOI:** 10.3389/fchem.2021.768248

**Published:** 2021-11-16

**Authors:** Xinyu Zhang, Yanling Ma, Jipeng Wan, Jia Yuan, Diqing Wang, Weiyi Wang, Xiao Sun, Qingwei Meng

**Affiliations:** ^1^ Department of Medical Oncology, Harbin Medical University Cancer Hospital, Harbin, China; ^2^ Department of Chemical and Biomolecular Engineering, National University of Singapore, Singapore, Singapore; ^3^ School of Chemistry and Pharmaceutical Engineering, Institute of Optical Functional Materials for Biomedical Imaging, Medical Science and Technology Innovation Center, Shandong First Medical University & Shandong Academy of Medical Sciences, Jinan, China

**Keywords:** biomimetic modification, nanomedicine, ferroptosis, diagnosis, cancer therapy

## Abstract

Ferroptosis, as a recently discovered non-apoptotic programmed cell death with an iron-dependent form, has attracted great attention in the field of cancer nanomedicine. However, many ferroptosis-related nano-inducers encountered unexpected limitations such as immune exposure, low circulation time, and ineffective tumor targeting. Biomimetic nanomaterials possess some unique physicochemical properties which can achieve immune escape and effective tumor targeting. Especially, certain components of biomimetic nanomaterials can further enhance ferroptosis. Therefore, this review will provide a comprehensive overview on recent developments of biomimetic nanomaterials in ferroptosis-related cancer nanomedicine. First, the definition and character of ferroptosis and its current applications associated with chemotherapy, radiotherapy, and immunotherapy for enhancing cancer theranostics were briefly discussed. Subsequently, the advantages and limitations of some representative biomimetic nanomedicines, including biomembranes, proteins, amino acids, polyunsaturated fatty acids, and biomineralization-based ferroptosis nano-inducers, were further spotlighted. This review would therefore help the spectrum of advanced and novice researchers who are interested in this area to quickly zoom in the essential information and glean some provoking ideas to advance this subfield in cancer nanomedicine.

## Introduction

### The Severe Problems in Treating Cancer and the Requirement of a New Application

From the report of the American Cancer Society, except cardiovascular diseases, tumors have become the second cause of death (Cortes et al., 2020). Similarly, according to the report from the World Health Organization, there were 9.6 million deaths caused by cancers in 2018 worldwide, which were 1/6 of the total deaths (Cortes et al., 2020). Although more and more researchers have devoted their studies toinnovating new cancer treatments, malignant tumor is affecting more patients with an increasing mortality rate (Altekruse et al., 2011). Cancer is still threatening people’s health severely. Nowadays, surgery, radiotherapy, chemotherapy, immunotherapy, and biotherapy are widely used in clinical cancer treatments. However, a high risk of relapse is also found in surgery because of the incomplete tumor cutting. The toxicity of radiotherapy and chemotherapy impairs patients’ normal organ functions, whereas biotherapy is too expensive to be afforded by most of the patients (Liu et al., 2019). In the past 10 years, immunotherapy, a promising cancer treatment (Yoon et al., 2018; Adams et al., 2019; Chu et al., 2019), has been impeded to be widely used in the clinic because of the narrow anticancer spectrum, the induction of potential autoimmune toxicity, and tumor escaping from the immune system (Adams et al., 2019; Sanmamed and Chen, 2019). Therefore, a safe and effective cancer treatment is yet to be invented to improve the effectiveness of cancer treatment and ameliorate the quality of patients’ life. In this case, nanotechnology can provide novel methods in treating cancers; nanomaterials can not only be used for loading and specifically targeting the chemotherapeutic drugs to the tumor sites but also have been included in other tumor treatment strategies.

### The Definition, Characteristics, and Association of Ferroptosis With Chemotherapy, Radiotherapy, and Immunotherapy for Enhancing Cancer Treatment Effects

Because of different morphologies, cell death is usually divided into three groups: apoptosis, autophagy, and necrosis (Gao and Jiang, 2018). Ferroptosis is a newly discovered cell death which is different from any other regulatory cell death in morphology, biochemistry, and genetics (Dixon et al., 2012). The cells in which ferroptosis occurs have smaller mitochondria, increased mitochondrial membrane concentration, reduced or disappeared mitochondrial cristae, and the rupture of the outer mitochondrial membrane. In addition, the inhibitors of apoptosis, autophagy, and pyrolysis cannot inhibit ferroptosis (Dixon et al., 2012; Yan et al., 2021). The sensitivity of cells toward ferroptosis is related to the regulation of multiple biological pathways, including iron metabolism, amino acid and glutathione (GSH) metabolism, and lipid metabolism (Stockwell et al., 2017).

Chemotherapy, one of the most common treatments for malignant tumors, prevents cancer cell proliferation and induces “cell death.” Chemotherapeutic drug resistance of tumor cells can be alleviated effectively by blocking ferroptosis. Some laboratories have reported that RSL3, the GSH peroxidase 4 (GPX4) inhibitor, can trigger ferroptosis *via* increasing reactive oxygen species (ROS) accumulation and lipid peroxidation (LPO) levels within the cells, thereby enhancing the antitumor efficacy of cisplatin (Sui et al., 2018; Zhang et al., 2020). In addition, erastin, a ferroptosis inducer, improves the antitumor effects of certain drugs such as temozolomide (Chen et al., 2015), cisplatin (Liu and Wang, 2019; Li et al., 2020), vemurafenib (Tsoi et al., 2018), and docetaxel (Zhou et al., 2019) in killing specific cancer cells.

Radiotherapy is a sufficient treatment in oncology which uses low linear energy–delivered ionizing radiation (such as X-ray or γ-rays) to kill or control malignant cells. Ionizing radiation releases free radicals directly or indirectly through the radiolysis of water to damage the cells (Bischoff et al., 2009). Several studies have shown that the ferroptosis inducers RSL3, erastin, sorafenib (SRF), and sulfasalazine synergistically enhance the radiation efficacy in the models of glioma, lung cancer, fibrosarcoma, melanoma, breast cancer, and cervical cancer (Pan et al., 2019; Lei et al., 2020; Ye et al., 2020).

Immunotherapy is a novel tumor treatment model. Programmed cell death protein 1 (PD-1) and programmed cell death ligand 1 (PD-L1) inhibitors are most widely used in this treatment to prevent PD-1 on the surface of T cells. In this case, cancer cells conduct “immune evasion” from T cells, restoring the T cell killing function toward cancer cells. Wang et al. found that with PD-1 inhibitors, the tumor volume of the tumor-bearing mouse was significantly reduced, and the lipid ROS was significantly increased at the same time (Wang et al., 2019). After applying the ferroptosis inhibitor lipoxstatin-1, the effect of PD-L1 inhibitors was reduced, which showed that ferroptosis plays an important role in immunotherapy.

The marketed drugs such as sulfasalazine (Chen et al., 2015; Ma et al., 2015; Roh et al., 2016), SRF (Louandre et al., 2013), and artemisinin and its derivatives (Roh et al., 2017; Chen et al., 2019) have been shown to induce ferroptosis in some tumors; therefore, they have great clinical value. At the same time, some of the malignant tumors such as human adrenal cortical carcinoma are very sensitive to the induction of ferroptosis, which means that ferroptosis may be directly used in the treatment of malignant tumors (Belavgeni et al., 2019). The progress of ferroptosis-related studies provides new strategies for tumor treatment sensitization and new ideas for developing new drugs.

### The Advantages of Biomimetic Nanomaterials and the Potential in Combining With Ferroptosis

A nanocarrier is a new type of drug delivery system at nanoscale. Because of the unique properties and easy modification, the nanocarrier is considered as a new generation of safe and specifically targeted drug carrier (Yang et al., 2016; Han et al., 2018). Nanocarriers have been successfully applied in diagnosis and precision therapeutic drug delivery for a better curative effect and reduced side effects due to their suitable size, easy modification, strong targeting ability, high cellular uptake, and good biocompatibility (Sumer and Gao, 2008; Melancon et al., 2011), which provide a new strategy to treat malignant tumors (Weissleder and Pittet, 2008; Barreto et al., 2011; Storm and Kiessling, 2011; Hrkach et al., 2012). However, some limitations have greatly hindered the clinical transformation of nanocarriers, for example, the toxicity as well as the degradation and metabolism of nanomaterials in the human body (Chou et al., 2014; Li et al., 2016; Shen et al., 2017). If nanomaterials enter the human body, there will be complex interactions between the drug carrier and the physiological environment, such as surface dissolution, protein adsorption, and/or non-specific cellular uptake which cause unfavorable tissue distribution, immune attack, and toxicity (Blanco et al., 2015). Therefore, the biocompatibility evaluation of nanocarrier materials has drawn more attention, such as blood compatibility, immune compatibility, and systemic toxicity. Nanomaterials with good biocompatibility can minimize the immune response of the body and reduce toxicity and side effects (Grossman and Mcneil, 2012). In tradition, the biocompatibility and biodistribution of nanoparticles can be partially optimized by nanoparticle surface modification (for example, PEGylation) (Xu et al., 2015; Moyano et al., 2016; Ni et al., 2021).

**FIGURE 1:**
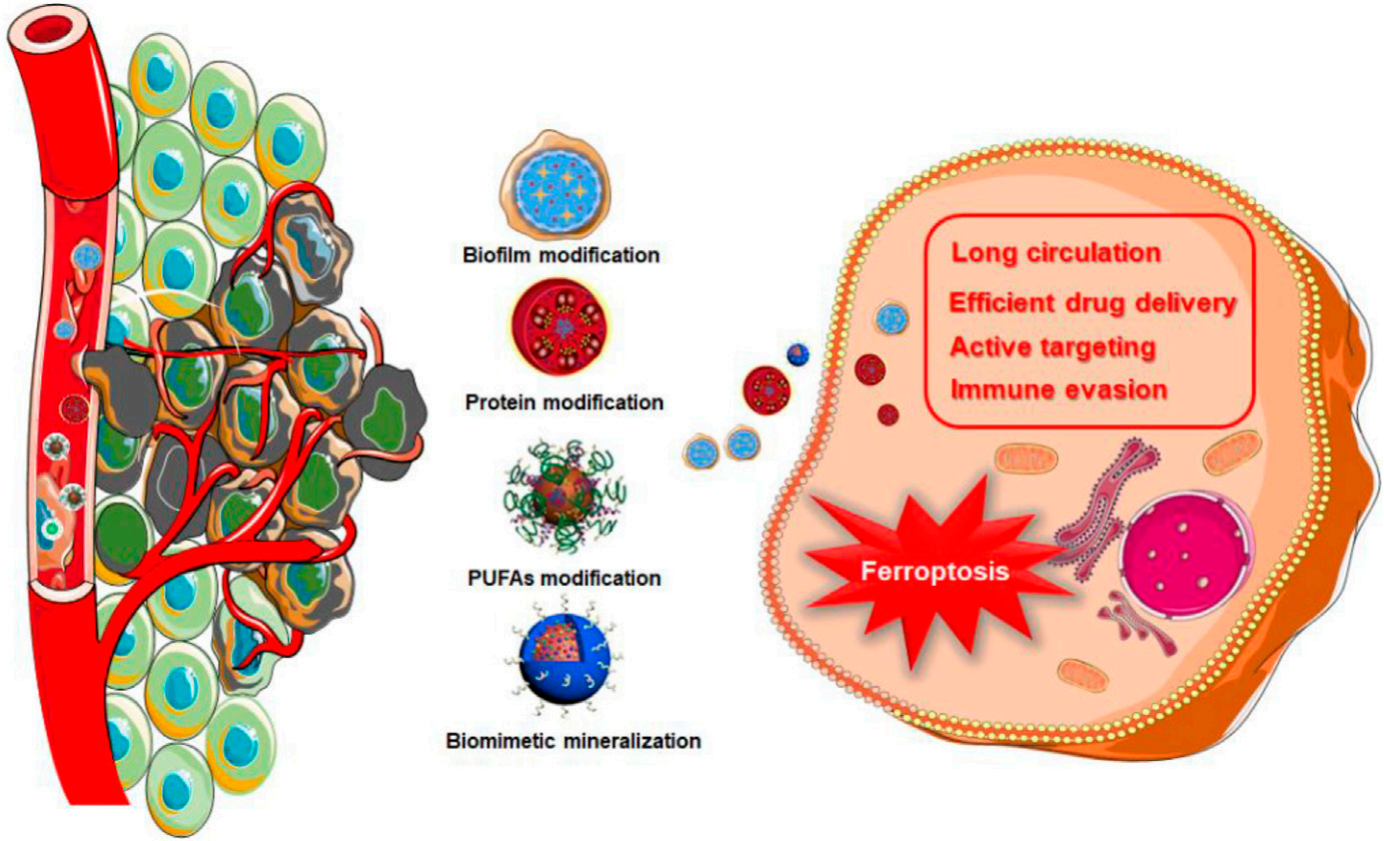
Schematic illustration of biomimetic nanomaterials for inducing ferroptosis.

In 1994, a s cientific research team headed by J. W. Bulte used horse spleen apolipoprotein as a raw material to synthesize a protein-encapsulated nano-single crystal superparamagnetic iron oxide as a magnetic resonance imaging (MRI) contrast agent (Bulte et al., 1994). This was the first time that the bionic technology was applied to a nanoassembly system. Since then, nano-biomimetic materials have entered a rapid developed stage. Biomimetic functionalization of nanoparticles could solve many problems in the physiological environment, endowing nanomedicines with better biological characteristics. For example, due to the antigenic diversity of the cell membrane, biomimetic modification provides a range of functions associated with the cell origin, including immune evasion, long circulation, efficient drug delivery, and active targeting (Zhen et al., 2019; Choi et al., 2020). As showed in Table 1, a lot of biomimetic nanomaterials are applied in cancer theranostics in recent years. Among them, biomimetic strategies for ferroptosis therapy can be divided into biofilm modification, protein modification, polyunsaturated fatty acids (PUFAs) modification, and biomimetic mineralization **(** Figure 1).

**TABLE 1:** Biomimetic nanomaterials for cancer theranostics.

System	Biomimetic Composition	Cancer Theranostics	Cancer Type	Ref
CCR2(+)-Fe-M1-Nvs	M1 macrophage membrane	Immunotherapy, ferroptosis	Breast cancer	Li et al. (2021)
FePSe_3_@APP@CCM	CT26 cell membrane	MR, PA, PTI/PTT, immunotherapy	Colorectal cancer	Fang et al. (2021)
Pa-M/Ti-NCs	Leukocyte membrane	MRI/immunotherapy, ferroptosis	Mutiple	Zhang et al. (2019)
Fe_3_O_4_-SAS@PLT	Platelet membrane	Immunotherapy, ferroptosis	Breast cancer	Jiang et al. (2020)
mFe(SS)/DG	4T1 cell membrane	Chemo-immunotherapy, ferroptosis	Breast cancer	Yang et al. (2021)
PNP-R848	Platelet membrane	Immunotherapy	Mutiple	Bahmani et al. (2021)
RB@Exo	Exosome membrane	Chemotherapy, PTT	Melanoma tumor	Shen et al. (2020)
EV-DNs	Grapefruit extracellular vesicles	Chemotherapy	Glioma	Niu et al. (2021)
DOX-PFP-CNs@PLGA/PM	Platelet membrane	PAI/PTT and chemotherapy	Breast cancer	Li et al. (2021)
GdTPP/ZnTPP	HeLa cell membrane	MRI, FI/PDT	Cervical cancer	Wang et al. (2020)
NPN	Bacteria membrane	PTT	Breast cancer	Li et al. (2020)
FGGZA	Glucose oxidase (GOx), BSA	PDT, PTT	Breast cancer	An et al. (2020)
BCFe@SRF	BSA	PDT, ferroptosis	Liver cancer	Wang et al. (2021)
P2K-ZnP-CRL-Bfr	Bacterioferritin	PDT, ferroptosis	Amelanotic melanoma	Cioloboc et al. (2018)
SRF@Hb-Ce6	Hb	PDT, ferroptosis	Breast cancer	Xu et al. (2020)
Hb-PDA-Fe@GOD@PEG-FA	Hb, GOD	PTT, PDT, starvation therapy	Mutiple	Yuan et al. (2021)
Tf-LipoMof@PL	Transferrin	Ferroptosis, pyroptosis	Breast cancer	Xu et al. (2021)
GHZD NCs	GOx	Immunotherapy	Breast cancer	Zhao et al. (2021)
ICG/AuNR@BCNP	Albumin	FLI, PAI, IRT, BLI, MRI, PET/PTT, PDT	Glioma	Yang et al. (2020)
AQ4N/GOx@ZIF-8@CM	GOx, HepG2 cell membrane	PDT	Liver cancer	Shao et al. (2021)
NMIL-100@GOx@C	GOx, 4T1 cell membrane	Ferroptosis, starvation therapy	Breast cancer	Wan et al. (2020)
AMSNs	Arginine	MRI/chemotherapy	Mutiple	Wang et al. (2018)
ACC@DOX.Fe^2+^-CaSi-PAMAM-FA/mPEG	Amorphous calcium carbonate	PDT	Mutiple	Xue et al. (2020)
GOx-MnCaP-DOX	MnCaP	MRI/CDT	Breast cancer	Fu et al. (2019)
OVA-Cu-HVs	Cu_3_(PO_4_)_2_, OVA	Immunotherapy	Lymphoma	Liu et al. (2019)
IO-LAHP	LAHP	MRI/PDT	Glioma	Liu et al. (2019)
LDL-DHA	ω-3 PUFA	Ferroptosis	Liver cancer	Ou et al. (2017)

Since Stockwell named ferroptosis for the first time in 2012, there have been a lot of research studies conducted to study its molecular mechanism and corresponding signal transduction pathways and try to find new and more efficient ferroptosis inducers for cancer treatments. So far, various genes, small molecules, and nanomaterials have been elucidated to possess the ability of inducing cell ferroptosis (Dixon et al., 2015; Louandre et al., 2015; Yu et al., 2015; Ma et al., 2016; Shen et al., 2018). However, the lack of endogenous iron and half-helical genes or molecular reagents cannot sufficiently improve the efficiency of the Fenton reaction. In addition, the selectivity of genes and small molecules is weak, and their adverse reactions also obstruct the clinical application transformation. In this case, the introduction of nanomedicine has brought bright future for developing new ferroptosis inducers in tumor-specific treatment. Among them, the unique physicochemical properties of nanomaterials can compensate the deficiencies of traditional drugs, such as low targeting efficiency, poor solubility, and severe adverse reactions, and can also introduce new properties such as magnetism, photothermal effects and electrochemical properties (Jiang et al., 2021). Most importantly, the ferroptosis nano-inducer can make up for the lack of endogenous iron and accelerate the ferroptosis process of tumor cells by improving the efficiency of the Fenton reaction.

Ferroptosis-induced nanomaterials can be mainly divided into two categories: one is iron-based nanomaterials and the other is non–ferrous-based nanomaterials. Due to the importance of iron in ferroptosis, iron-based compounds with abundant iron ions, such as iron oxide nanoparticles (IO NPs), are the most popular nanomaterials in ferroptosis application–related research. In addition, there are also some iron-free nanomaterials which can induce cancer cell death by cooperating with endogenous iron. For example, Kim et al. created an iron-free, ultrasmall α-melanocyte–stimulating hormone and polyethylene glycol–modified silica nanoparticles which are effective ferroptosis inducers for tumor suppression and targeted therapy (Kim et al., 2016). This review mainly summarized the latest developments of biomimetic nanomaterials in the field of the development trends of ferroptosis-related tumor treatment and some considerations about the principles, advantages, and limitations of these bionic strategies.

## A Variety of Bionic Nanomaterials Are Used for Ferroptosis-Mediated Tumor Treatment

### Biofilm Modification

With the inspiration of the natural biological system, biofilm bionics has drawn scientists’ attention (Fang et al., 2017). In recent years, various biofilms have been extracted to modify nanoparticle surfaces to produce multifunction bionic nanoparticles which are composed of nanoparticles as the core and natural biofilms as the shell. The nanoparticle core endows the bionic nanoparticle high selectivity, and biofilms also make it natural. By comparing with the attachment of nanomaterial functional groups, biofilm bionic nanomaterials are easy to prepare with better biocompatibility to lower immune rejection and toxicity. Therefore, biofilm bionic nanomaterials show great application prospects in medicine.

Leukocytes are immune cells which maintain the normal functions of the immune system, including macrophages, neutrophils, eosinophils, basophils, and lymphocytes (Xuan et al., 2015). The bionic nanomedicine of the leukocyte membrane can be constructed for targeted delivery of antitumor drugs by utilizing the mutual recognition between the leukocyte and tumor surface antigen–antibody (Molinaro et al., 2020). The receptors on the neutrophils’ surface can interact with chemokines and adhesion factors in the tumor microenvironment to enrich the drug (Zhang et al., 2019; Sun et al., 2020). Kang et al. reported that NMNP-CFZ, a neutrophil membrane biomimetic nanoparticle, was able to block tumor metastasis by capturing tumor cells from the blood stream and specifically targeting the tumor microenvironment prior to metastasis (Kang et al., 2017). Macrophages are non-specific immune cells that can bind specifically to tumor surface antigens and have chemotactic properties to the tumor microenvironment. Bionic modification of the macrophage membrane can reduce the clearance rate of drug carriers *in vivo*, prolong the systemic circulation time, enrich drugs in the tumor site, significantly inhibit the tumor growth, and improve therapeutic effect (Zhao et al., 2018). Lymphocytes can be divided into NK cells, T cells, and B cells. NK cells are innate immune cells and can secrete cytokines to activate T cells and regulate immune responses (Wehner et al., 2011). The NK cell membrane has the function of tumor targeting and inducing M1-type polarization of macrophages (Deng et al., 2018).

**FIGURE 2:**
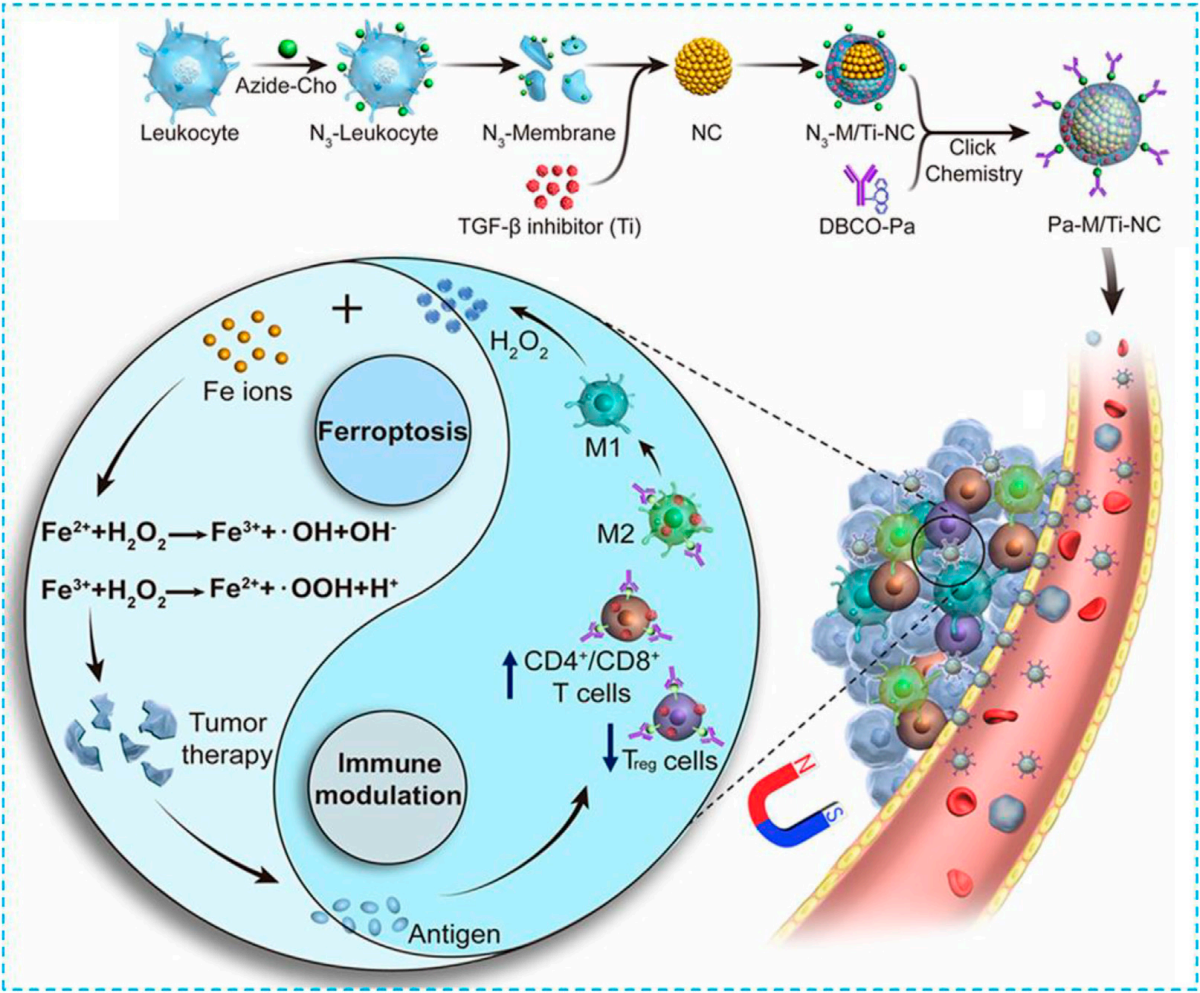
Schematic illustration of the NMNP-CFZ nanoplatform for ferroptosis/immunomodulation synergism in cancer. Reprinted with permission from Ref. (Lyu et al., 2020) Copyright 2019 American Chemical Society.

In 2019, Xie and coworkers constructed a magnetosome Pa-M/Ti-NCs; the core of the magnetosome was a superparamagnetic and controllable Fe_3_O_4_ nanocluster (NCS) which was made *via* a one-pot hydrothermal approach (Wan et al., 2020). This specific structure can not only be used for MRI and magnetic targeting but also delivers a large amount of Fe ions to conduct the Fenton reaction and induce ferroptosis **(** Figure 2 **)**. At the same time, the previously designed leukocyte membrane by using N3 covered NCS with a typical membrane protein (such as CD44 and CD45) to prolong circulation time and promote the loading of Ti, a TGF-β inhibitor within the membrane, and the coupling with the dibenzocyclooctyne-modified PD-1 antibody. After getting into the tumor, Pa and Ti worked together to create an immunogenetic microenvironment to elevate the concentration of H_2_O_2_ within the polarized M1 macrophage which promoted the Fenton reaction with iron ions released from NCS. The produced hydroxyl free radicals (•OH) then induced ferroptosis of tumor cells, and the exposed tumor antigens enhanced immunogenicity of the microenvironment further. In the B16F10 xenograft model, the signal of free NCS without membrane coating was detected at the tumor site for up to 6 h. The 24-h signal did not satisfy because of the fast clearance. In comparison, the leukocyte-coated NCS could significantly inhibit phagocytosis of macrophages to increase circulation and enhance EPR, and therefore, a better antitumor effect could be achieved.

Macrophages, a subclass of leukocytes, are important effector cells in innate immunity which have great functional plasticity during tumor development and metastasis. More and more studies have proved that macrophages are used in cancer treatment for better therapeutic effects (Wan et al., 2020). Inspired by the macrophage-based cancer therapy and exosome-like drug delivery, in 2021, Li and his colleagues designed an exosome; the M1 macrophages which were transfected by CCR2 plasmids were coincubated with Fe_3_O_4_ nanoparticles and further extruded into exosome-like nanovesicles (denoted as CCR2 (+)-Fe-M1-NVS) to act as a nano-Fenton reactor and M2 repolarization inducer to restrict lung metastasis synergistically (Li et al., 2021). Both *in vitro* and *in vivo* experiments have found that CCR2 (+)-Fe-M1-NVS was similar to the mature macrophages which accumulated at tumor metastases through the CCR2-CCL2 (C–C chemokine-receptor 2 and -ligand 2, respectively) axis. Fe_3_O_4_ nanoparticles and M1-related factors (such as H_2_O_2_) were known as catalysts of the Fenton reaction to promote the ferroptosis of tumors and jointly induce the repolarization of macrophages, therefore stimulating tumor-specific immune responses. At the same time, the repolarization of M2 macrophages induced H_2_O_2_ accumulation by upregulating T-cell–stimulating factors, activated T cells, and synergistically induced ferroptosis by releasing interferon-γ, thereby inhibiting the uptake of cystine by tumor cells, leading to LPO. By investigating the cell coculture system and the 4T1 mouse xenograft model, the targeted therapy and synergistic cytotoxicity were verified.

Platelets are small fragments of the cytoplasm derived from megakaryocytes in the bone marrow. Platelets have a relatively low content in the blood, but they are super-important for body hemostatic function. When there is a wound, platelets can be quickly recruited to the wound to activate both internal and external coagulation systems for hemostasis (Senturk, 2010). Because of the fast response to vasculature damage, the recognition of circulated tumor cells, and the specific binding to the damaged area, platelet-coated nanoparticles have been widely used in cancer treatment (Zhang et al., 2018). At the same time, endothelial reticulum endocytosis can also be reduced, and the immune escape of nanomedicine can be increased to extend the blood circulation of medicine and finally promote the efficacy of the drugs (Hu et al., 2015; Dehaini et al., 2017; Chen et al., 2019; Wang et al., 2019).

Yang et al. reported the production of a magnetic nanoparticle Fe_3_O_4_-SAS@PLT (Jiang et al., 2020). Sulfasalazine (SAS) is used to treat rheumatism which can inhibit not only inflammatory cell migration and the IκB kinase pathway but also cysteine intake to suppress the tumor growth and induce ferroptosis (Roh et al., 2016). The SAS activity has been proved by a clinical tumor model when the concentration is high. SAS was uptaken by magnetic nanoparticles and camouflaged by the platelet membrane; this composite was used for cancer treatment by inducing the ferroptosis effect. As a ferroptosis inducer, the synergy between Fe_3_O_4_ and SAS reduced the SAS dose significantly. *In vitro*, the concentration of Fe_3_O_4_-SAS@PLT for maximum inhibition was lower than Fe_3_O_4_-SAS, which indicated that PLT coating enhanced cytotoxicity because of the high binding affinity between PLT and the CD44 receptor on tumor cells (Ye et al., 2019). At the same time, PLT coating increased the cellular uptake of the nanoparticle composite. Therefore, platelet membrane coating can escape from the immune system, specifically bind to tumors, and maximize the uptake of the ferroptosis-inducible nanoparticle by the tumor which showed a great potential in treating tumor metastases.

In recent years, cancer cell–coated nanocarriers have drawn researchers’ attention. This type of biomimetic membrane can camouflage nanomedicine as cancer cells to deliver drugs to the tumor site *via* mutual cancer cell recognition and adhesion to achieve effective cancer treatment (Zhang et al., 2019; Zhen et al., 2019). The design and application of cancer cell–coated nanocarriers have become hot topics for cancer diagnosis and treatment. Cancer cells could endow nanoparticles the ability to target the tumor specifically and escape from the immune system to improve the accumulation of nanoparticles in the tumor (Zhang et al., 2019; Zhen et al., 2019). Yang et al. reported a novel nanoplatform mFe (SS)/DG as ROS–ferroptosis–glycolysis regulators for the anticancer immune cycle (Yang et al., 2021). Constructed by Fe^3+^ and disulfide (-S-S-)-containing organic ligand, the nanosystem with a suitable surface and structure can effectively load GOx and doxorubicin (DOX) and can be further coated by the 4T1 cell membrane to decrease immune clearance and realize efficient tumor targeting. This nanoplatform can consume intracellular GSH *via* -S-S- bond and catalyze glucose to generate excessive ROS in tumor sites, improve cascade-amplified ferroptosis, and elicit immunogenic cell death in combination with DOX. By combining tumor metabolism and immune function, the biomimetic nanoplatform provides a potential antitumor strategy.

**FIGURE 3:**
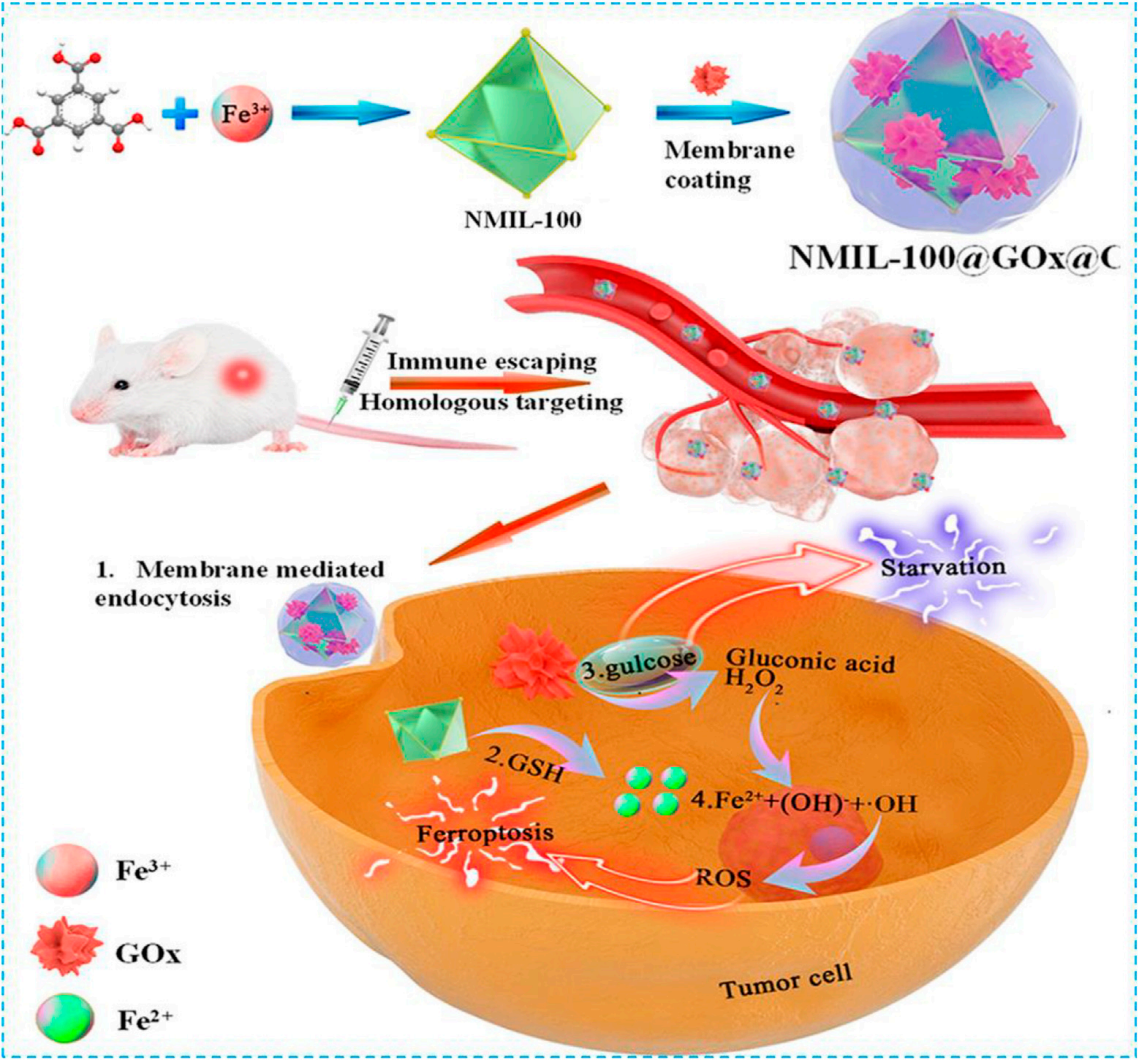
Schematic illustration of the preparation and cascade processes for cancer therapy of NMIL-100@GOx@C. Reprinted with permission from Ref. (Wan et al., 2020). Copyright 2020 American Chemical Society.

Tang et al. designed a nanoreactor which was composed of cancer cell–coated organic iron framework and GOx (Wan et al., 2020). In this nanoreactor, GOx catalyzed the production of H_2_O_2_ from glucose for the induction of the ferroptosis effect. When the nanoreactor reached the tumor site, MOF collapsed because of the release of Fe^2+^ from high concentration of GSH reduced Fe^3+^ worked with GOx to produce H_2_O_2_ **(** Figure 3 **)**; then the Fenton reaction between H_2_O_2_ and Fe^2+^ released •OH to promote the ferroptosis effect. The fluorescent intensity of the cancer cell–coated nanoreactor NMIL-100@RhB@C was as twice as NMIL-100@RhB and could be endocytosed easier by 4T1 cells. Furthermore, the detection of the antiphagocytosis ability of NMIL-100@RhB@C and NMIL-100@RhB against macrophages (Raw 264.7) indicated that fluorescent intensity increased twice in NMIL-100@RhB incubated Raw264.7. NMIL-100@RhB@C–injected mice showed higher fluorescent intensity on the tumor site, especially within the tumor site, than NMIL-100@RhB–injected mice which proved that the cancer-coated nanoreactor can specifically bind to the tumor site. During nanoreactor transportation, the cancer cell coating can protect the nanoreactor from proteinase and immune system degradation, and therefore, high concentration of GOx can reach and be endocytosed by the tumor. Overall, these cascade reactions provided a safer and more efficient way to inhibit the tumor growth by triggering both ferroptosis and tumor cell starvation.

The cell membrane biomimetic nanoprobe is a new type of probe which actively identifies specific tumor cells *via* the EPR effect or molecular targets to shift tumor therapy from the tissue and organ level to the molecular level. At the same time, the cell membrane nanoprobe restricts the clearance of the reticuloendothelial system and prolongs the circulation time in the body significantly. Therefore, it has unique advantages in tumor-targeted precision therapy. Although the design of biomimetic cell membranes has bright prospects, new problems have also existed. For example, how to stabilize the biological effects of heterogeneous white blood cells in the body is unignorable. Whether the cancer cell membrane will express oncogenes and whether its biological safety can be guaranteed are also yet to be studied. After the cancer cell membrane enters the body, the changes of the internal immune system can be triggered. At the same time, some issues may be encountered, such as how to produce abundant various cell membrane nanoprobes efficiently, simplify the production process with reduced cost, and how to ensure the activity and stability of biofilm materials which will be the direction of future research. In addition, how to improve interactions between the biofilm and the tumor site to improve its recognition ability also should be considered because there may be limited opportunities for nanomaterials *in vivo* to establish contacts with the tumor before clearance.

### Protein Modification

Proteins are big molecules with biological functions. The protein nanocarrier system is composed of different structures and functions of animals and plants or recombinant proteins and drugs which have good biocompatibility, biodegradability, low antigenicity, high stability, and drug loading capacity and are easy to make (Lee et al., 2016; Sandra et al., 2019). There are a large amount of free amino acids on the protein surface, and they can be modified with photothermal molecules, fluorescent dyes, chemotherapeutic drugs, image probes, photosensitizers, and many other small functional molecules which make the protein nanoparticle a good nanocarrier to prolong anticancer drug loading and circulating for improved cancer imaging and treatment (Wang et al., 2015). For example, due to its good biocompatibility and stability, bovine serum albumin (BSA) is often used for nanomaterials’ surface modification. In the process of stripping lamellar MoS_2_, an effective stripping agent and stabilizer, BSA can achieve the large-scale preparation of ultrathin MoS_2_ nanosheets under ultrasonic conditions. In another study, as a template, BSA is expected to enable the programmed preparation of TMD QDs to improve their biocompatibility and stability (Wang et al., 2020). In addition, in ferroptosis-related studies, iron ion–based proteins (hemoglobin, ferritin, etc.) are often involved in enhancing the level of iron ions, or some functional proteins such as GOx are often associated with H_2_O_2_ production.

**FIGURE 4:**
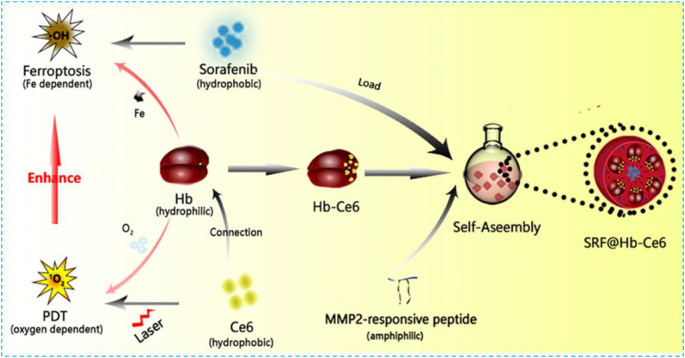
Schematic illustration showing the components of SRF@Hb-Ce6, the synthesis process, and the combined PDT and ferroptosis therapy. Reprinted with permission from Ref. (Xu et al., 2020). Copyright 2020 American Chemical Society.

Hemoglobin (Hb) is one of the most well-studied natural and red endogenous proteins to transfer oxygen in the blood vessels (Liu et al., 2018). Because of high oxygen capability, it is always used to donate oxygen in photodynamic therapy. Hb has four heme groups, which contain irons and can be used as iron supplements in the ferroptosis effect (Xu et al., 2020). These iron ions can be released in the presence of H_2_O_2_ and produce •OH *via* the Fenton reaction (Xu et al., 2020). Li and colleagues constructed a nanoplatform SRF@Hb-Ce6 *via* loading SRF to the Hb-coated photosensitizer Ce6 **(** Figure 4 **)** (Xu et al., 2020). Both oxygen and irons in Hb brought opportunities for the combination of PDT and ferroptosis effect. In this nano platform, high oxygen concentration promoted PDT efficiency significantly; SRF and intrinsic iron in Hb greatly elevated lipid peroxide production and suppressed GPX4 to induce strong ferroptosis effects. This nanoplatform could also enhance T-cell infiltration at the tumor site and the release of IFN-γ to downregulate SLC3A2 and SLC7A11 which increased the sensitivity of tumors toward ferroptosis. By comparing with free Ce6, the fluorescent image of SRF@Hb-Ce6 showed a stronger intensity at the tumor site which indicated SRF@Hb-Ce6 had specific binding.In the *in vivo* study, SRF@Hb-Ce6-treated tumor-bearing mice had smaller tumor sizes and higher survival rates. Therefore, SRF@Hb-Ce6 can promote both PDT and the ferroptosis effect, which showed us a promising strategy in treating cancer safely via the combination of two treatments.

**FIGURE 5:**
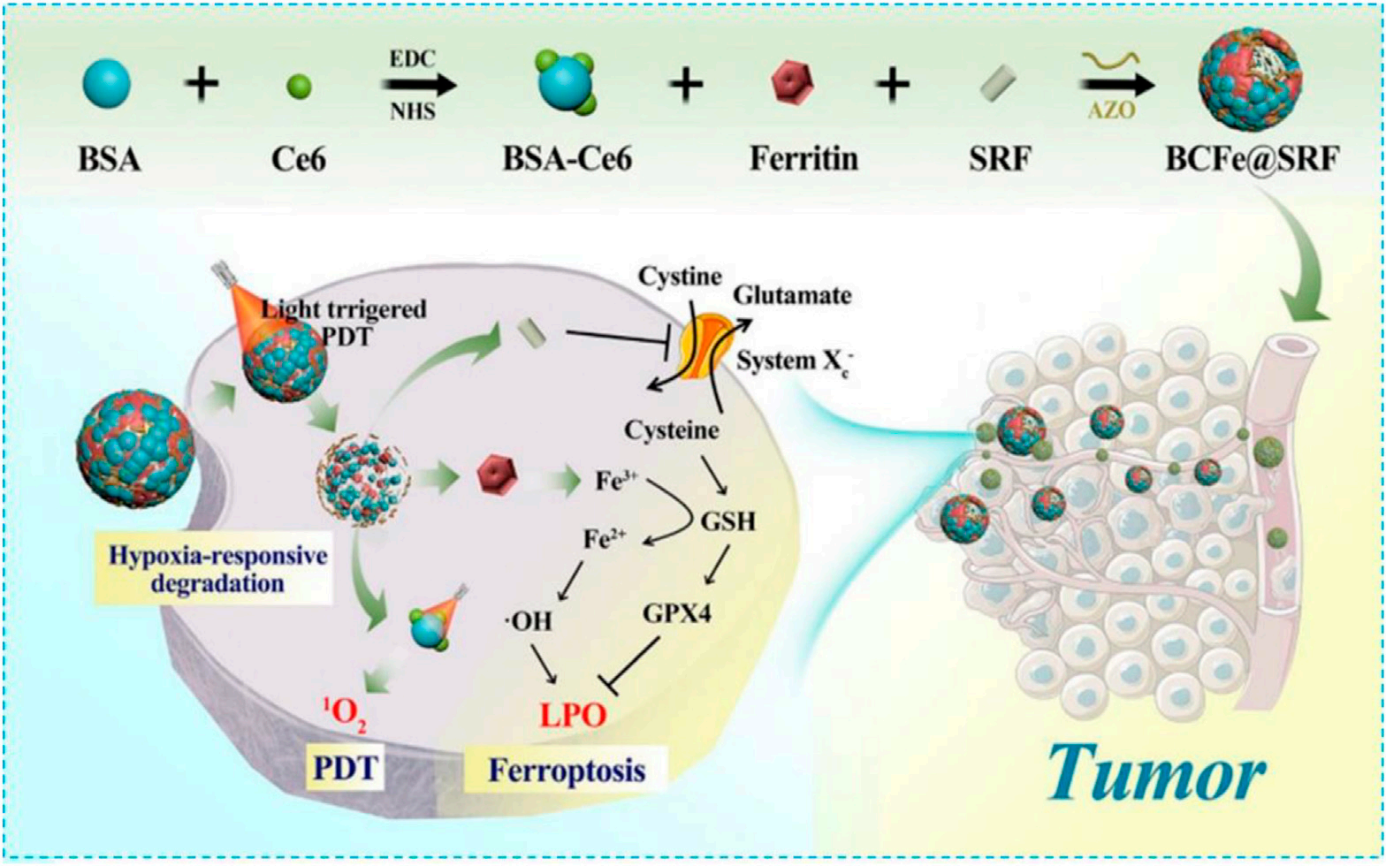
Schematic illustration showing fabrication procedures and antitumor mechanisms of the BCFe@SRF nanoreactor for the synergistic PDT and ferroptosis therapy. Reprinted with permission from Ref. (Wang et al., 2021). Copyright 2021 The Author(s).

Ferritin is a protein which can store irons. It has been identified by overexpressed TFR1 as a new type of missile platform (Liang et al., 2014; Truffi et al., 2016). In addition, the stored irons, released from ferritin intracellular autophagy, can induce ferroptosis easily (Ensor et al., 2002; Miyamoto et al., 2003; Phillips et al., 2014; Gao et al., 2016; Hou et al., 2016; Keshet et al., 2018; Yang et al., 2021). Therefore, with intrinsic iron absorption capacity and difficult-to-identify characteristics, ferritin has more advantages than inorganic materials or MOF materials in treating ferroptosis. In 2021, Wang et al. covalently linked BSA-Ce6 (Ce6 coupled BSA) and ferritin by azobenzene (Azo) to fabricate a new hypoxia-responsive device BcFe@SRF for SRF loading which provided an unparalleled opportunity for the synergistic treatment of high-efficiency photodynamic (PDT) and ferroptosis **(** Figure 5 **)**. The designed BcFe@SRF had suitable particle size, stable dispersion, and excellent tumor-homing performance. Importantly, in a hypoxia environment, BcFe@SRF could be gradually degraded and release BSA-Ce6, ferritin, and SRF for triggering PDT, the iron-catalyzed Fenton reaction, and destroying tumors’ antioxidant defenses, respectively. Apart from the PDT effect, studies have also found that BcFe@SRF-mediated low-oxygen laser irradiation could not only promote the production of LPO but also depleted intracellular GSH and reduced the expression of GPX4. In summary, the BcFe@SRF nanoreactor uses a variety of ways to promote the accumulation of intracellular ROS and exerts significant antitumor effects both *in vitro* and *in vivo*.

Transferrin (TF) is a glycoprotein to carry irons and mediate iron endocytosis when it binds to the transferrin receptor (TFR) (Torti and Torti, 2013). TF has been proved to be expressed in various types of cancer cells. (Johnsen et al., 2017) The upregulated TFR level on the surface of cancer cells enhances TF-mediated iron endocytosis and can therefore be a sufficient and specific cancer treatment (Weed et al., 1963; Daniels et al., 2012). More Fe^3+^ can be captured by extra TF to further accelerate TFR endocytosis. Shen et al. constructed a nanosphere TF-LipoMof@PL by coating the TF-modified pH-sensitive lipid membrane on piperlongumine (PL)-loaded MOF (Xu et al., 2021). The lipid membrane could promote TF-mediated iron endocytosis to provide a prerequisite of the ferroptosis effect. As a ferroptosis inducer, PL provided H_2_O_2_ for producing ROS via the Fenton reaction. *In vitro*, the TF-precipitated nanosystem showed higher tumor cytotoxicity. ICP-MS–measured intracellular iron levels showed TF-LipoMof@PL-applied cancer cell had the highest iron level, which means TF could increase iron endocytosis. The *in vivo* study had similar results, among 4T1-transplanted mice with a high TFR expression level; TF-LipoMof@PL-treated cells had the highest ROS level and induced the strongest iron effect. Therefore, TF-LipoMof@PL had the best anticancer effect.

GOx is a natural enzyme; H_2_O_2_ can be released when it oxidizes dextrose to gluconic acid in the present of oxygen (Wang et al., 2016). The tumor growth is highly dependent on glucose supply; tumor cells will be too starving to grow once glucose supply is stopped (Zhang et al., 2018; Xie et al., 2019; Yang et al., 2019). GOx-inducing glucose oxidation could lower the glucose level, which makes it attractive in cancer treatment. This treatment is the so-called starvation therapy (Fu et al., 2018; Yu et al., 2018; Zhang et al., 2018; Fu et al., 2019; Hanjun et al., 2019; Xie et al., 2019). At the same time, a high level of H_2_O_2_ was produced during GOx catalyzation which can cooperate with the iron-based nanomaterial to promote ferroptosis (Bankar et al., 2009; Fu et al., 2018; Hanjun et al., 2019). An et al. designed a 4,4′-azonzenecarboxylic acid (Azo)–BSA–modified zeolitic imidazolate framework (ZIF) nanoplatform with Fe^3+^-gallic acid and GOx encapsulation (designated as FGGZA) (An et al., 2020). FGGZA achieved sustained Fe^2+^/H_2_O_2_ supply and decreased pH and O_2_ levels, which can significantly improve the ferroptosis reaction microenvironment. In a hypoxic microenvironment, azo achieved charge reversal, leading to selective tumor aggregation based on efficient cell internalization activated by hypoxia. This reasonably designed biomimetic nanosystem will present great potential in clinical transformation of ferroptosis tumor treatment.

**FIGURE 6:**
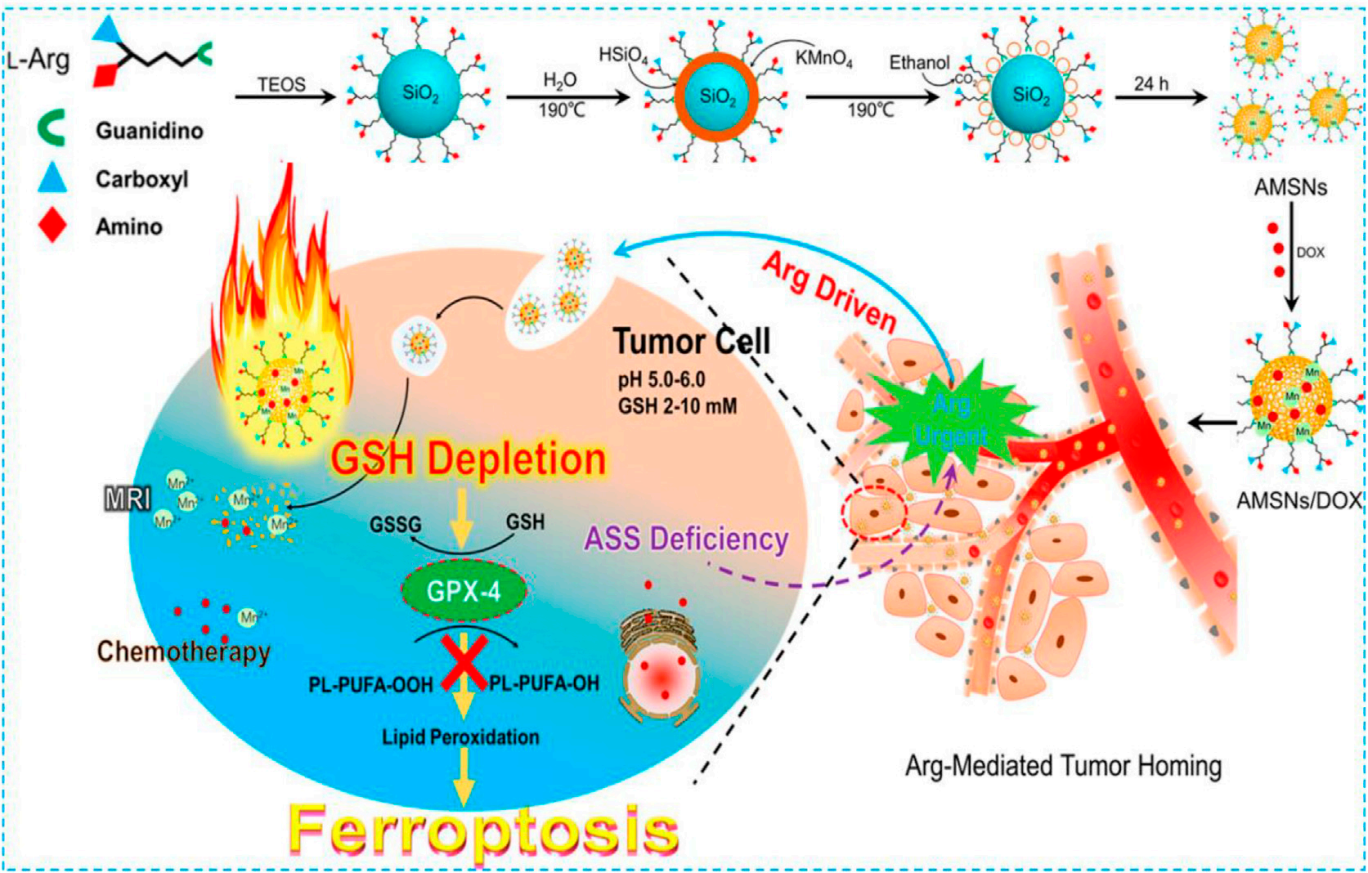
Schematic illustration of the preparation and ferroptosis-induced cancer therapy of AMSNs. Reprinted with permission from Ref. (Wang et al., 2018). Copyright 2018 American Chemical Society.

As the main component of protein, amino acid modification is also considered to be an effective biomimetic method. Due to the lack of arginine (Arg) succinate synthase (ASS) in a variety of tumors, such as breast cancer, renal cell carcinoma, melanoma, and hepatocellular carcinoma, the tumor cells themselves cannot produce Arg (Ensor et al., 2002; Phillips et al., 2014; Keshet et al., 2018). Considering that the consumption of Arg at tumor sites will lead to the demand for Arg-modified nanomaterials, Arg is considered a targeted part of tumor diagnosis and treatment. In 2018, Wang et al. used a one-pot method to fabricate an Arg-rich manganese silicate nanobubble (AMSN)–based ferroptosis inducer which was endowed the high-efficiency GSH depletion capacity to inactivate GPX4 to induce ferroptosis (Figure 6 **)** (Wang et al., 2018). In this case, Arg acted as a surface-capping ligand to provide ideal water dispersibility, biocompatibility, and tumor-homing ability. In comparison with traditional nanoparticles, the ultrathin surface layer and nanobubble structure of Arg significantly improved the rate of GSH depletion in AMSNs and inactivated tumor cells by GPX4. Fer-1 and DFO can remarkably reduce the toxicity of AMSNs. As the concentration of AMSNs and the incubation time increased, the protein expression level of GPX4 decreased and the activity of GPX4 reduced. Then, fluorescein dichlorodiacetate (DCFH-DA) proved that as AMSNs depleted GSH, the level of reactive oxygen species in the cell increased. The level of LPO was monitored by the specific probe C11-BODIPY581/591. The fluorescence intensity of AMSNs was stronger than that of the control group, and it was proportional to the concentration and the incubation time. These results indicated that ferroptosis plays a crucial role in AMSN-induced cell death. In addition, the degradation of AMSNs during GSH depletion caused Mn ions and drug release to enhance the contrast of T1-weighted MRI and the effect of chemotherapy. Different from the iron-based nanomaterials which induce ferroptosis through the Fenton reaction or manganese-based nanomaterials which only provide auxiliary functions to consume GSH in tumor treatment, AMSN can directly induce ferroptosis *in vitro* and *in vivo*, and therefore, high-efficiency tumor targeted therapy can be achieved.

In the process of protein nanomaterial–induced ferroptosis, apart from the high structural stability, excellent biocompatibility, and wonderful biodegradability of protein nanocarriers, the natural properties of proteins can be used, such as the iron-rich protein, or the protein improves the transport efficiency of iron which can increase the concentration of endosomal iron ions and improve the effect of ferroptosis further. However, these protein nanoparticles still have shortcomings. For example, the size of protein nanoparticles cannot be precisely controlled if they are prepared by covalent or non-covalent binding due to uncontrollable self-assembly behavior. Some proteins are difficult and expensive to be obtained, such as ferritin. More attention should be paid to maintain protein activity in appropriate temperature and buffer solution to avoid inactivation during the process of the experiment. At the same time, immunogenicity brought by the enzyme itself should also be concerned. Due to the large size and the fixed three-dimensional structure of some proteins, it is more difficult to modify the surface of some nanomaterials than long-chain polymers (e.g., PEG and PVP). In addition, the macrophage system uptakes nanomaterials significantly because of the wide size distribution and easy aggregation which reduce the concentration of protein nanoparticles in the tumor site. Furthermore, many excellent inorganic nanomaterials cannot directly interact with proteins, which limits the application of inorganic nanomaterials in tumor diagnosis and treatment. Therefore, it is still necessary to continue to explore protein nanoparticles with precise and controllable size to overcome the existing drawbacks listed before.

### Polyunsaturated Fatty Acid Modification

**FIGURE 7:**
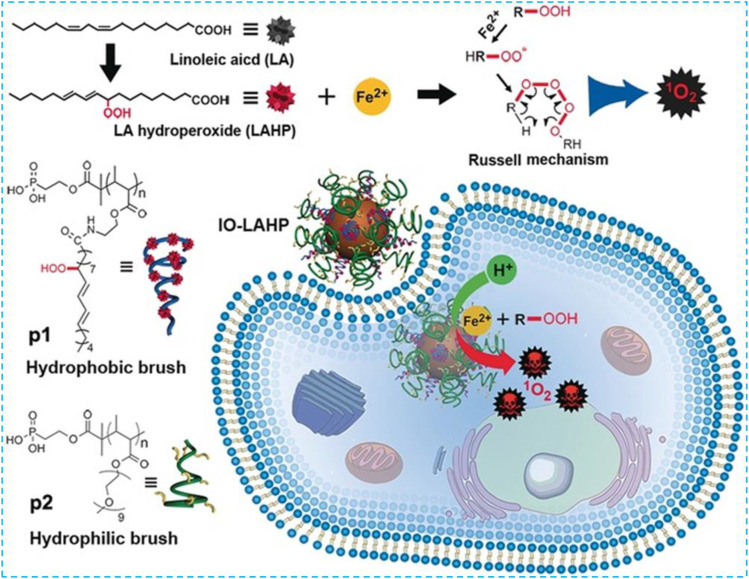
Preparation and ferroptosis-mediated cancer therapy of IO-LAHP. Reprinted with permission from Ref. (Zhou et al., 2017). Copyright 2017 Wiley-VCH.

PUFAs are important components of the phospholipid bilayer which is crucial in maintaining the fluidity of the cell membrane. Excess PUFAs will be oxidized into ·OH by Fe^2+^ via the Fenton reaction. These ·OH can further oxidize PUFAs in a chain reaction to produce a large amount of LPO and finally induce cell ferroptosis. In 2017, Zhou et al. used hydrophobic linoleic acid hydrogen peroxide (LAHP) and hydrophilic oligoethylene glycol to modify phosphate groups on the surface of IO NPs to develop a Fenton reaction–based nanosystem (Figure 7) (Zhou et al., 2017). Under this circumstance, IO NPs with a diameter of 22 nm were used as carriers of LAHP polymers with surface anchoring groups. Hydrophilic polymers grafted with oligoethylene glycol units were used as end-capping molecules on the surface of IO NPs; therefore, IO-LAHP nanoparticles possessed water dispersibility, proton permeability, and biocompatibility. At the same time, H^+^ could penetrate the polymer brush to dissociate Fe^2+^ from the surface of the IO-LAHP nanoparticles as an iron source and realize the on-demand release of Fe^2+^ by the nanosystem under tumor acidic conditions. As one of the main products of LPO, LAHP was decomposed into free radicals and ^1^O_2_ by catalytic ions (such as Fe^2+^and Ce^4+^) through the Russell mechanism (Miyamoto et al., 2003; Miyamoto et al., 2006). After intravenous injection of IO-LAHP nanoparticles in mice, the overall tumor growth was significantly inhibited. By evaluating the efficiency of IO-LAHP nanoparticles in producing activated ^1^O_2_ and in cancer treatment *in vitro* and *in vivo*, LAHP-modified IO NPs can treat cancer effectively through the non-photodynamic process when ^1^O_2_ is produced in engineering biochemical reactions.

Similarly, the addition of exogenous PUFA DHA to tumor cells provides a new possible nanomaterial construction which induced ferroptosis by promoting LPO because cancer cells tend to actively take up lipoproteins to meet the lipids’ demands for rapid membrane turnover (Favre, 1992). At the same time, the lipoprotein platform can transport lipids in the plasma naturally, and it is also a particularly suitable carrier for omega-3 PUFAs (ω-3 PUFAs) (Gotto et al., 1986). Based on this knowledge, in 2017, Ou et al. uniformly incorporated w-3 PUFA docosahexaenoic acid (DHA) into a low-density lipoprotein (LDL) to construct LDL-DHA nanoparticles (Ou et al., 2017). After treating using LDL-DHA, both rat and human liver cancer cells underwent significant LPO, GSH depletion, and the deactivation of the lipid antioxidant GPX4. The antitumor effect of LDL-DHA nanoparticles was evaluated in mice with HepG2 xenografts. PBS, LDL-TO and LDL-DHA, and Fer-1 or LDHA + Fer-1 were injected intratumorally to treat tumor-bearing mice, and the growth of the tumor treated by the low-density lipoprotein DHA was significantly inhibited. The combination of Fer-1 and LDL-DHA can remarkably antagonize the GPX4 activity, level of LPO, and tumor volumes which were treated by LDL-DHA alone.

So far, many studies have proved that ω-3 PUFAs have cytotoxicity toward various cancer cell cultures in a dose-dependent manner (Lindskog et al., 2006; Lim et al., 2009). However, the dosage required to induce anticancer effects in the culture is hard to achieve (Conquer and Holub, 1998) through dietary intake which may explain the inconsistent results from the treatment of established tumors through dietary intake of ω-3 PUFAs (Noguchi et al., 1997; Swamy et al., 2008; Gleissman et al., 2011). Poor solubility in water and easy forming of emboli make direct intravascular injection of ω-3 PUFAs inviable (Mellor and Soni, 2001). Even in cell culture experiments, organic solvents such as ethanol or dimethyl sulfoxide are needed to dissolve the ω-3 PUFAs in the growth media. Therefore, nanoparticles should be good tools to treat tumors. Ferroptosis can be regulated during unsaturated fatty acid metabolism; unsaturated fatty acid–loaded nanoparticles also have great prospects in this field. However, considering the uncertainty of the degree of ferroptosis induced by a single unsaturated fatty acid, more combinations of nanomaterials should be considered, such as with small-molecule ferroptosis inducers or iron-based nanomaterials to design more advanced nanomaterials in the treatment of tumor ferroptosis.

### Biomimetic Mineralization

Biomineralization is a common process in which inorganic ions are combined with biological macromolecules to generate hard biomaterials in organisms. Organisms (bacteria, plants, animals, etc.) with different compositions can generate minerals in the body, endowing them on specific biological functions. The biological mineralization is a highly controlled process that exists naturally and is regulated by the genetics of the organism. It can achieve the precise control of the assembly of the crystal shape and structure from the molecular level to mesoscopic level, attracting more attention of scientists in the biomedical field. For example, the nanodrug slow-release system based on biomineralization has received extensive attention in cancer treatment due to easy preparation and modification, good biocompatibility, and biodegradability; therefore, a multifunction nanoplatform related to biomineralization has been developed for a variety of biomedical applications (Srivastava et al., 2018).

CaCO_3_ is one of the most common inorganic materials found in nature. Because of the low cost, bio-absorbility, and good biocompatibility, CaCO_3_ has attracted wide attention of many researchers and scientists (Mao et al., 2016). However, high crystallization made CaCO_3_ degrade slowly in the biological environment, which would impede drug release and therefore block the progression of its application as a drug release system (Maleki Dizaj et al., 2015). The development and application of the amorphous calcium carbonate (ACC) nanostructure has finally solved these intrinsic limitations. Compared with the CaCO_3_ crystal, ACC nanoparticles have a higher energy level and can be hydrolyzed easily within cells. After a series of experiments (Min et al., 2015; Dong et al., 2018; Wang et al., 2018), the advantages of the ACC nanodrug carrier have been demonstrated. Zhao’s group obtained an ACC-based, pH-sensitive drug delivery system by co-condensing DOX and a calcium precursor which can only release DOX under an acidic tumor microenvironment to kill cancer cells (Zhao et al., 2015). Furthermore, organic-modified ACC can further improve the bio-functionality and biocompatibility of nanocomposites without changing its original clinical advantages.

**FIGURE 8:**
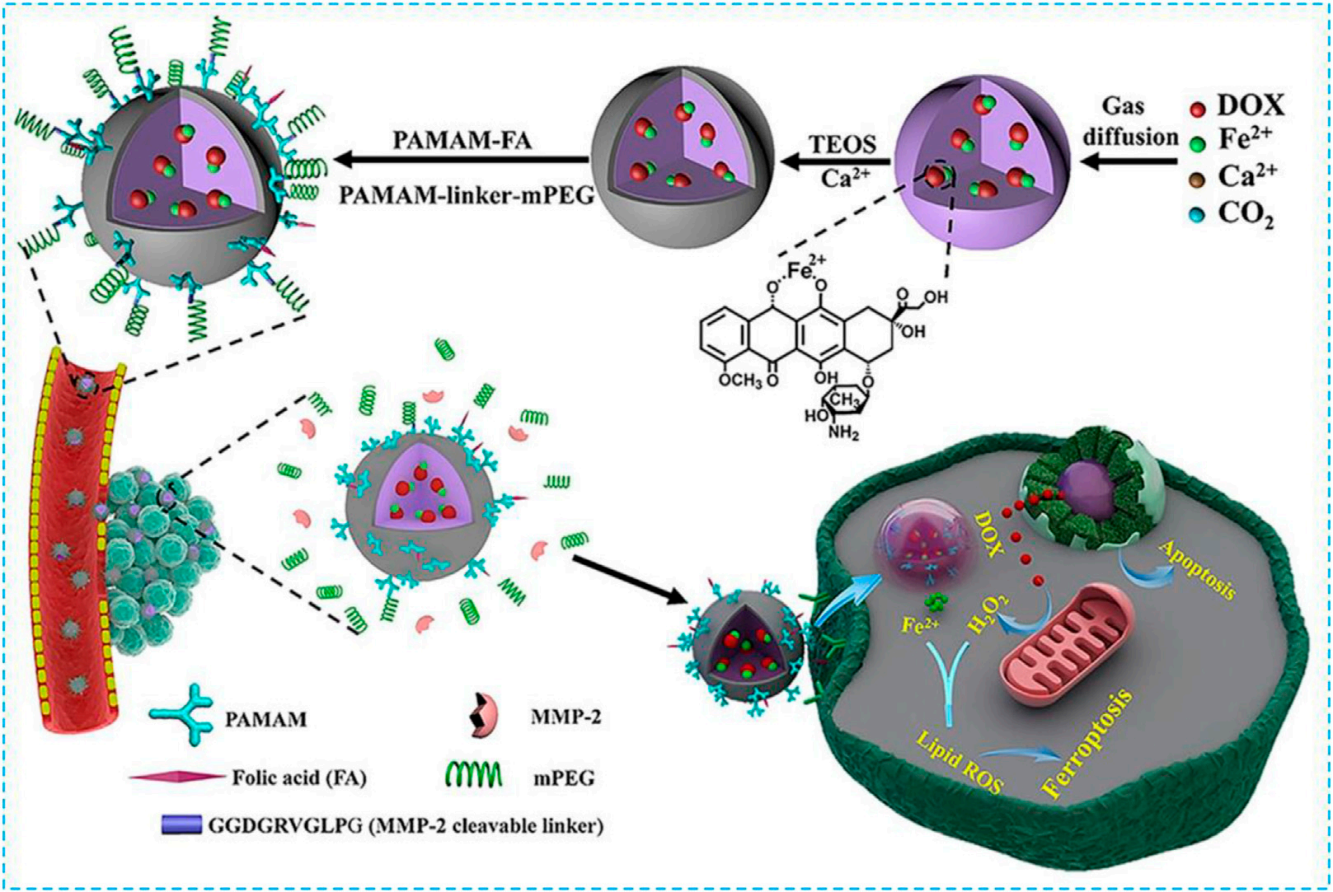
Synthesis scheme of ACC@DOX.Fe^2+^-CaSi-PAMAM-FA/mPEG and its complementary ferroptosis/apoptosis-based therapeutic action. Reprinted with permission from Ref. (Xue et al., 2020). Copyright 2020 The Authors.

Luo et al. chelated DOX with Fe^2+^ and co-condensed with the calcium precursor to construct an ACC-coated Fe^2+^-DOX core in a one-step process based on amorphous ACC which can be used for tumor targeting and ferroptosis treatment (Figure 8 **)** (Xue et al., 2020). In this case, the combination of DOX and Fe^2+^ can not only be loaded sufficiently into ACC but also minimize the sensitivity of ferrous towards oxidative stress before it releases from the host cell so that the biodegradable ACC can work with DOX and Fe^2+^ synergistically. The thin CaSi layer covered at the core is coupled with PAMAM to endow the specific tumor binding and provide a balance between circulatory life and tumor-specific uptake. DOX can be released within the cancer cells when acid-induced ACC degradation has occurred and H_2_O_2_ produced Fe^2+^-promoted ferroptosis. This nanocomposite has showed a strong ferroptosis effect and will be used in clinic.

Compared with synthetic nanomaterials, these biosynthetic nanominerals have good physiological stability, biodegradability, and biological activity and simple and economical synthesis process, and only need a simple bionic water system. Therefore, the design of a variety of nano-biominerals by combining biomineralization technology and ferroptosis for tumor treatment has broad application prospects in tumors. However, biomimetic-mineralized nanomaterials also faced many problems. For example, some biomimetic-mineralized nanomaterials about urinary calculus, such as calcium oxalate, calcium phosphate, and uric acid, may not degrade well *in vivo* and require further manual intervention to remove them. Because the biomimetic mineralization is highly ordered, it is difficult to control the reaction process during the artificial preparation, and some reaction conditions are relatively demanding. In addition, biomimetic mineralized nanomaterials synthesized *in vitro* may also cause immune reaction in a certain degree when applied *in vivo*. In addition to genetic regulation and other biological methods to control the synthesis of biomimetic mineralized materials, scientists still need to make unremitting efforts to further explore the generation mechanism and action properties of biomimetic mineralization.

## Conclusion, Fresh Perspective, and Future Directions

Due to the antiapoptotic effect of tumor cells caused by overexpression of apoptosis-inhibiting proteins and multidrug resistance, apoptosis-based treatment strategies cannot achieve satisfactory therapeutic effects. Ferroptosis, a novel non-apoptotic programmed cell death, has become a hot topic in cancer research and has gradually attracted wide attention in the field of cancer nanomedicine. The emergence of biomimetic nanomaterials brings new opportunities for the clinical translation of ferroptosis-triggered nanomaterials. Synthetic nanoparticles are combined with natural biological materials to create biomimetic nanomedicine inspired by nature. These nanomaterials not only have the adjustability and flexibility of synthetic materials but also have the functionality and biocompatibility of natural materials, thus giving nanomaterials many advantages such as good versatility and a long *in vivo* cycle. Moreover, some components of biomimetic materials can enhance ferroptosis more effectively. This review summarized the latest research studies in the field of biomimetic nanomaterials to trigger ferroptosis of tumor cells and enhance tumor therapy. We hope that this review will provide new ideas and insights for the application of biomimetic nanomaterials in the biomedical field.

Despite the rapid progress of ferroptosis-based cancer therapeutics, the potential clinical applications of ferroptosis nanomedicine still face many challenges. For example, there are significant differences in the sensitivity of ferroptosis toward various species and tissues. And the sensitivity of ferroptosis inducers (SRF, erastin, etc) is also diverse. Therefore, it is of great significance to search for biological indicators that can reflect the sensitivity of cells and individuals to ferroptosis and to develop new ferroptosis inducers to improve the level of tumor diagnosis and treatment. In addition, several studies have shown that the combination of different therapies (e.g., PDT and PTT) with ferroptosis therapy through nanotechnology can achieve better therapeutic effects, further overcoming the barriers of ferroptosis treatment (Wang et al., 2021; Yuan et al., 2021).

There are still a lot of difficulties in the large-scale application of biomimetic nanoparticles to trigger ferroptosis due to the difficulties existed in the preparation, storage, and application process of some biomimetic nanoparticles. Theoretically, while bionic methods can effectively reduce the response of the body, there is still a lack of long-term tracking validation in *in vivo* experiments which need more exploration by excellent scientists. Because of the deficiency of the iron content for some ferroptosis nano-inducers, iron ion loading and the controlled release capacity of ferroptosis nano-inducers should be intensified to improve the distribution of iron in the cancer cells and thus improve anticancer efficiency. Although biomimetic modification can endow nanomedicine good biocompatibility, some of the nanomaterial components are difficult to be degraded or may generate toxic products after degradation. Therefore, in the process of designing biomimetic nanodrugs, the safety of the proposed component should be considered.

In conclusion, as a novel tumor treatment and multidisciplinary research, ferroptosis induced by biomimetic-based nanotumor therapy has a high therapeutic efficiency in killing cancer cells and reducing the toxicity and side effects on normal cells/tissues. Compared with the traditional cancer treatment, it shows obvious advantages and clinical transformation potential. After the listed limitations of this field have been fully addressed in future, the progress of cancer biomedicine discovery will be promoted greatly and benefit more cancer patients.
